# Seroprevalence of HIV in pregnant women in North India: a tertiary care hospital based study

**DOI:** 10.1186/1471-2334-7-133

**Published:** 2007-11-15

**Authors:** Swati Gupta, Richa Gupta, Sarman Singh

**Affiliations:** 1 Division of Clinical Microbiology, Department of Laboratory Medicine, All India Institute of Medical Sciences, New Delhi-110029. India

## Abstract

**Background:**

Estimating the seroprevalence of HIV in a low risk population such as pregnant women provides essential information for an effective implementation of AIDS control programmes, and also for the monitoring of HIV spread within a country. Very few studies are available from north India showing the current trend in HIV prevalence in the antenatal population;which led us to carry outthis study at a tertiary care hospital in north India

**Methods:**

Blood samples from pregnant women attending antenatal clinics at the All India Institute of Medical Sciences, New Delhi were collected after informed consent and pre-test counseling. The samples were tested for HIV antibodies as per the WHO guidelines, over a period of four years from January 2003 to December 2006.

**Results:**

Of the 3529 pregnant women tested in four years, 0.88% (CI 0.5 – 1.24) women were found to be HIV seroreactive. Majority of the seroreactive pregnant women (41.9%) were in the age group of 20–24 years followed by the 30–34 yrs (25.8%) and 25–29 years (22.6%) age group. The mean age of the HIV positive women was 24.9 years (SD ± 1.49 yrs). The HIV seroprevalence rates showed an increasing trend from 0.7% (CI 0.14 – 2.04) in 2003–2004 to 0.9% (CI 0.49 – 1.5) in 2005–2006. This prevalence rate indicates concern, as Delhi and its adjoining states are otherwise considered as 'low prevalence states'.

**Conclusion:**

Seroprevalence of HIV infection was found to be increasing in the last four years amongst pregnant women of North India. These findings are in contrast to the national projections.

## Background

India is categorized as a low prevalence nation for HIV with a seroprevalence rate of less than 1% among the adult population [1]. The country experienced a sharp increase in the estimated number of HIV infections, from a few thousand in the early 1990s to around 5.2 million adults and children living with HIV/AIDS in 2005 [2]. In view of our large population pool of one billion plus, a mere 0.1 per cent increase in the prevalence rate will raise the number of persons living with HIV by over half a million. In India, the predominant mode of HIV transmission is through heterosexual contact [3], therefore, unsuspecting women are at high risk of getting the infection.

The trend of new or incident infections, especially in young people who have recently become sexually active, is the most sensitive marker to track the course of the HIV epidemic. Unfortunately, incidence is hard to measure directly, but prevalence in young women is an indirect but useful proxy [4]. Accordingly, HIV data from antenatal women has been used to monitor trends in the general population and to predict the seroprevalence in young children [5,6]. In children below the age of 15 years mother to child transmission is by far the most significant route of transmission of HIV infection. While heterosexual contact is the commonest mode of spread of the virus in this country, perinatal transmission accounts for 4% of the total HIV infection load in India [7]. As the HIV-positive women in India are increasing in number, consequently the number of babies acquiring HIV infection in the perinatal period is also expected to increase if the infection goes undetected during pregnancy. Therefore, screening of pregnant women at an early stage of pregnancy may help in prompt counseling and therapy, thereby reducing the risk of transmission to the child.

To the best of our knowledge, only a few studies on HIV prevalence in antenatal women are available from north India, and infact none indicating the current trend in seroprevalence from this area. Hence, we undertook this study to determine the rate and trends of HIV seroprevalence among pregnant women attending antenatal clinics at the All India Institute of Medical Sciences, New Delhi, which is a tertiary care hospital of India and caters to patients from most states of North India.

## Methods

### Setting

This study was carried out in the Microbiology Division of the Department of Laboratory Medicine at the All India Institute of Medical Sciences, New Delhi. This institute is a tertiary care referral hospital.

### Patients and period of study

Pregnant women registered at the antenatal clinics of this hospital are routinely advised to undergo HIV and hepatitis B screening after pre-test counseling and informed consent. Our laboratory caters these laboratory services to all such patients, and tests are carried out as per the guidelines laid down by the National AIDS Control Organization (NACO), India [8]. It is important to mention that even though patients with other ailments are referred to this hospital from all parts of India, the antenatal clinic does not register pregnant women from other states, except complicated cases. This policy is because obstetrical services are freely available in all peripheral centers also. While registering in the antenatal clinic, stay of more than 5 years is considered an eligibility criteria. The results were collected from all pregnant women tested in this laboratory and no selection bias was observed. The findings were analyzed over four years from January 2003 to December 2006. All the tests were done after due patient consent and in accordance with the institutional ethical guidelines.

### Specimen

Five ml venous blood sample was collected in a sterile plain container from all pregnant women who came for testing. Blood was allowed to clot for 30 minutes at room temperature (25–30°C) and serum was separated after centrifugation at low speed. The serum samples were then stored at 4°C and were used within 48 hrs.

### Serology

HIV antibodies were tested by the three ELISA/Rapid/Supplemental tests protocol as per the guidelines laid down by the World Health Organization (WHO Testing strategy III) and positive test result disclosed only after post-test counseling of the patients. Antibodies to HIV (1&2) were tested initially with an ELISA kit (*Adaltis*™, Italy) and repeatedly reactive samples were tested by a rapid test (Tri-dot, *J Mitra & Co*, India). The reactive sera were further confirmed by Western Blot (BioRad, USA).

### Statistical analysis

The data were analyzed using the Chi-square tests. The confidence interval for the prevalence estimates was calculated using STATA-9 statistical software.

## Results

Data were collected and analyzed from a total of 3,529 pregnant women who were tested during the period of four years from January 2003 to December 2006. The mean age of the women attending antenatal clinics and advised HIV screening was 26.4 years (95% CI = 1.82). Majority of the pregnant women (41.7%) were in the age group of 25–29 years followed by 20–24 years (34.6%), 30–34 years (17.7%), 35 yrs or more (4.2%) and least in the 15–19 yrs (1.6%) age group.

Overall, HIV-1 antibodies were detected in 0.88% (31 out of 3,529) of the subjects [95% CI 0.5–1.24]. No pregnant female was found seroreactive for HIV-2 antibodies. Amongst the seropositive women, the majority (41.9%) were aged 20–24 years followed by the age group of 30–34 yrs (25.8%) and 25–29 yrs (22.6%). Only three (9.7%) pregnant women below 20 years of age were detected HIV positive while no woman above 34 years was found positive. The mean age of the HIV positive women was 24.9 years (SD ± 1.49 yrs). The youngest HIV positive female was aged 18 years while the oldest was 32 years.

A year-wise analysis showed that in 2003 only 137 pregnant women were tested for HIV, most probably, because the national policy of mandatory antenatal screening was not in place at the institute by that time. After our institutional guidelines were modified, the number of patients screened for HIV increased to 289 in 2004, 1553 in 2005 and 1550 in 2006. The HIV seroprevalence rates showed an increase from 0.7% (CI 0.14–2.04)in 2003–2004 to 0.9% (CI 0.49–1.5) in 2005–2006 [Table 1].

**Table 1:** Year-wise prevalence rates of HIV in pregnant women at a tertiary care centre in north-India

Year	Total tested	HIV positives	% positivity (95% CI)
2003–2004	426	3	0.70 (0.14–2.04)
2005	1553	14	0.90 (0.49–1.5)
2006	1550	14	0.90 (0.49–1.5)

Total	3529	31	0.88 (0.5–1.24)

HIV seropositivity was highest at 1.2% (CI 0.7–2.02) in the younger pregnant females in the 18–24 years of age-group as compared to the older women (0.7%; CI 0.4–1.17) of 25–34 years of age group [Table 2]. However, statistical analysis showed that this age-specific prevalence was not significant (p > 0.5). The year-wise age-standardized prevalence rates showed that while there was little change in prevalence rates from 1.3% (CI 0.1–4.7) in 2003–2004 to 1.2% (CI 0.67–2.07) in 2005–2006 in the 15–24 years age group, a marginal rise in prevalence was seen in the 25–34 yrs age group from 0.4% (CI 0.01–2.2) in 2003–2004 to 0.7% (CI 0.41–1.26) in 2005–2006.

**Table 2:** Age-standardized year-wise trends in HIV-prevalence in pregnant women at a tertiary care hospital in north India

	**Overall**			**2003 & 2004**			**2005 & 2006**		
**Age (yrs)**	Total Tested	HIV +ve	% positives (95% CI)	Total Tested	HIV +ve	% positives (95% CI)	Total Tested	HIV +ve	% positives (95% CI)

**15–24**	1281	16	1.2* (0.7–2.02)	151	2	1.3* (0.1–4.7)	1130	14	1.2* (0.67–2.07)
**25–34**	2099	15	0.7 (0.4–1.17)	250	1	0.4 (0.01–2.2)	1849	14	0.7 (0.41–1.26)

*The HIV prevalence rates were higher in the 15–24 year age-group, but not significant (p > 0.5)

## Discussion

India's socio-economic status, traditional social ills, cultural myths on sexuality and a huge population of marginalized people make it extremely vulnerable to HIV/AIDS [9]. Since the first case report in 1986 in Chennai in South India, HIV has spread rapidly from urban to rural areas and from high-risk groups to the general population [1-3]. In a country of over one billion population and 5.2 million HIV positive adults in the 15–49 years age group, India is now faced with multiple HIV-epidemics [10]. Heterosexual contact remains the major mode of transmission, thereby resulting in a growing population of HIV infected women (38% in 2005) [2].

The present study, even though not representing the general population, provides clear insight of an increasing trend of HIV seroprevalence at the rate of 0.88% among pregnant women in India. These findings have significance because as per the official data from India's National AIDS Control Organization (NACO) [1], Delhi and the adjoining north-Indian states are categorized as low prevalence areas for HIV. In these states the HIV prevalence rates among the vulnerable population groups are reported to be below 5% and in women attending antenatal clinics less than 1%. Only the states of Maharashtra, Tamil Nadu, Manipur, Andhra Pradesh, Karnataka and Nagaland are categorized into high prevalence states where the HIV seroprevalence is more than 1% in women attending antenatal clinics. The NACO sentinel surveillance data for the state of Delhi reported HIV prevalence of 0.25% in 2003, 0.38% in 2004 [1] and 0.25% in 2005 [10]. Therefore; our data indicate a different scenario where the prevalence rate was found to be significantly higher (0.88%) than the NACO data. One reason for this difference between the data of NACO and ours could be surveillance methodologies. While ours was a hospital based study over a period of 4 years, the NACO utilizes sentinel surveillance system. Both the systems have their own limitations. Beside the data from NACO, as there is no other study reported from this part of India, for comparison of our findings.

Our results also become significant because the HIV seroprevalence rate has reached almost 0.9% in pregnant women which is very close to the cut-off value of 1% used to categorize a state as low or high prevalence area. This finding of rising HIV seropositivity rate in antenatal women in an area previously considered 'low prevalence region' of India, could indicate a increasing trend of HIV transmission in the general population of this region, in contrast to the claims of 'a contained epidemic of HIV in India' by national agencies. While analyzing the trend of seroprevalence, we found a rise in the HIV seroprevalence rate from 0.7% in 2003–2004 to 0.9% in 2005–2006. Even though the change in prevalence rate was statistically insignificant (p > 0.5) due to non-comparable number of women included in the first and fourth year of the study, the trend indicated concern.

The prevalence of HIV seropositivity was highest (1.2%) in the newly sexually active pregnant females belonging to the 18–24 years age-group. These findings are similar to a study among antenatal women in western India by Ukey *et *al [11] in which the authors found that HIV seroprevalence increased from 1.24% in 2002–2003 to 1.45% in the year 2003–2004. This study also reported that the most affected age group was 18–24 years followed by the older age groups [11]. It has been well substantiated that most women in India are infected with HIV through their husbands [3,12]. This holds true for both rural as well as urban settings. Our results showed only a marginal decrease in prevalence rates in young, newly sexually active females (15–24 yrs) from 1.3% in 2003–04 to 1.2% in 2005–2006. This slight dip in prevalence could be a result of effective awareness programmes and education regarding HIV especially in young adults after the implementation of National AIDS control programme (NACP-II, 1999–2006). The NACP II sought to shift focus from raising awareness to changing behavior through interventions in high risk groups. Intervention programmes such as HIV awareness and safe sex education are usually focused on young adults and our data show a favorable impact of such programmes. Therefore, it is understandable why over a period of 4 years the HIV seroprevalence showed a decline in young adult mothers. Since the impact of such programmes will have marginal effect in older couples, our data were on expected lines. Even Pallikadavath *et al *[13] showed that HIV awareness among north Indian married women was higher in the 15–24 years age group as compared to older women. In a recent study published by Kumar *et al *[4], a decreasing trend in HIV prevalence has been reported in South Indian pregnant women aged 15–24 years from 1.7% to 1.1% within a period of four years from 2000 to 2004 after antenatal counseling and awareness programmes were initiated and increasing propagation of safe sex was started. These awareness programmes have difficult pervasiveness in older women and hence, it is not surprising that seroprevalence rates continued to increase over the study period. It is also possible that these women were infected in past (not incidence rate) but were detected during the study period when the HIV testing for these women was made available. Moreover, these women have a higher reproductive history with higher rates of exposure to risk factors like surgical interventions during successive pregnancies and higher risk of sexually transmitted infections (STI's). This has been further compounded by lower levels of education, low access to health care facilities and higher rates of promiscuous activities of their husbands before the aggressive awareness programmes were launched. It is well known that women are less likely to visit a public antenatal clinic if they are older, have high parity, are illiterate, or are poor [5,6] making this a vulnerable group. Lastly, in our study, we found only 149 pregnant women in the age group of 35 yrs and above, who were tested for HIV over a period of four years of study. Though we could not find any HIV positive pregnant women in this age group, yet this number of pregnant women is very small to comment upon.

## Conclusion

Our study indicates an increasing trend of HIV prevalence in northern India. Even though, our study population is not representative of whole India because of ours being a hospital based study with limited sample size, the data show an increasing trend of HIV spread in housewives and pregnant mothers. This will directly transform into a high perinatal transmission and a reciprocal increase in pediatric AIDS cases. Therefore, it may be recommended that even though the curative treatment for HIV is not available at present we can minimize, if not prevent, the pediatric HIV infection by early screening of pregnant mothers for HIV followed by perinatal short term chemotherapy, safe delivery practices and modified infant feeding.

## Competing interests

The author(s) declare that they have no competing interests.

## Authors' contributions

SS was responsible for conceptualizing the study, arranging diagnostic services and facilities and critically reviewing the manuscript. SG analysed the data and prepared the draft of manuscript and RG helped SG in data analysis and manuscript preparation. All authors read and approved the manuscript.

## Pre-publication history

The pre-publication history for this paper can be accessed here:



## References

[B1] National AIDS Control Organization (NACO), Government of India Annual Report 2002–2004. http://www.nacoonline.org.

[B2] UNAIDS-AIDS Epidemic Update: December 2006. http://www.data.unaids.org.

[B3] Srikanth P, John TJ, Jeyakumari H, Babu PG, Mathai D, Jacob M, Cherian AM, Ganesh A, Zachariah A (1997). Epidemiological features of acquired immunodeficiency syndrome in southern India. Indian J Med Res.

[B4] Kumar R, Jha P, Arora P, Mony P, Bhatia P, Millson P, Dhingra N, Bhattacharya M, Remis RS, Nagelkerke N (2006). International Studies of HIV/AIDS ISHA) investigators. Trends in HIV-1 in young adults in south India from 2000 to 2004: a prevalence study. Lancet.

[B5] Zaba B, Boerma T, White R (2000). Monitoring the AIDS epidemic using HIV prevalence data among young women attending antenatal clinics: prospects and problems. AIDS.

[B6] Boerma JT, Ghys PD, Walker N (2003). Estimates of HIV-1 prevalence from national population-based surveys as a new gold standard. Lancet.

[B7] NACO Guidelines for the prevention of mother to child transmission of HIV. http://www.naco.nic.in/pmtct.html.

[B8] Baveja UK HIV Antibody Testing with special reference to HIV-1. HIV Testing Manual, Laboratory diagnosis, Biosafety and Quality Control.

[B9] Singh S (2003). Food crisis and AIDS: Indian perspective. Lancet.

[B10] HIV/AIDS epidemiological Surveillance & Estimation report for the year NACO, April 2006. http://www.nacoonline.org.

[B11] Ukey PM, Akulwar SL, Powar RM (2005). Seroprevalence of human immunodeficiency virus infection in pregnancy in a tertiary care hospital. Indian J Med Sci.

[B12] Gangakhedkar RR, Bentley ME, Divekar AD, Gadkari D, Mehendale SM, Shepherd ME, Bollinger RC, Quinn TC (1997). Spread of HIV Infection in Married Monogamous Women in India. JAMA.

[B13] Pallikadavath S, Jayachandran AA (2005). Stones RW. Women's Reproductive Health, Sociocultural Context and AIDS Knowledge in Northern India. J Health Manag.

